# PTBP3 promotes malignancy and hypoxia‐induced chemoresistance in pancreatic cancer cells by ATG12 up‐regulation

**DOI:** 10.1111/jcmm.14896

**Published:** 2020-01-27

**Authors:** Jun Ma, Li Weng, Yiping Jia, Bingyan Liu, Shaoqiu Wu, Lei Xue, Xiang Yin, Aiwu Mao, Zhongmin Wang, Mingyi Shang

**Affiliations:** ^1^ Department of Interventional Radiology Tongren Hospital Shanghai Jiao Tong University School of Medicine Shanghai China; ^2^ Shanghai Key Laboratory of Signaling and Diseases Research School of Life Science and Technology Tongji University Shanghai China; ^3^ Department of interventional radiology Ruijin Hospital Shanghai Jiao Tong University School of Medicine Shanghai China

**Keywords:** autophagy, chemoresistance, hypoxia, pancreatic cancer, polypyrimidine tract‐binding protein 3

## Abstract

Pancreatic ductal adenocarcinoma (PDAC) tumours exhibit a high level of heterogeneity which is associated with hypoxia and strong resistance to chemotherapy. The RNA splicing protein polypyrimidine tract‐binding protein 3 (PTBP3) regulates hypoxic gene expression by selectively binding to hypoxia‐regulated transcripts. We have investigated the role of PTBP3 in tumour development and chemotherapeutic resistance in human PDAC tissues and pancreatic cancer cells. In addition, we determined the sensitivity of cancer cells to gemcitabine with differential levels of PTBP3 and whether autophagy and hypoxia affect gemcitabine resistance in vitro. PTBP3 expression was higher in human pancreatic cancer than in paired adjacent tissues. PTBP3 overexpression promoted PDAC proliferation in vitro and tumour growth in vivo*,* whereas PTBP3 depletion had opposing effects. Hypoxia significantly increased the expression of PTBP3 in pancreatic cancer cells in vitro. Under hypoxic conditions, cells were more resistance to gemcitabine. Knockdown of PTBP3 results in decreased resistance to gemcitabine, which was attributed to attenuated autophagy. We propose that PTBP3 binds to multiple sites in the 3′‐UTR of ATG12 resulting in overexpression. PTBP3 increases cancer cell proliferation and autophagic flux in response to hypoxic stress, which contributes to gemcitabine resistance.

## 1 INTRODUCTION

Pancreatic ductal adenocarcinoma (PDAC) is the fourth leading cause of cancer‐related mortality worldwide despite a low incidence rate.^1^, ^2^ Moreover, less than 20% of patients with PDAC survive for 1 year and only 5%‐7% survive for five years.^3^ These low survival figures are partly owing to tumour heterogeneity and the rapid development of chemoresistance.^4^ In particular, patients can respond poorly to gemcitabine which is the first‐line treatment against PDAC.^5^, ^6^ In clinical trials, gemcitabine has been found to give better results than fluorouracil in overall survival and the alleviation of disease‐related symptoms.^7^ However, resistance to gemcitabine can develop in sensitive tumours within a few weeks predominately because the vascular‐deficient dense tumour microenvironment of PDAC does not allow adequate drug penetration.^8^, ^9^ Furthermore, cells that can propagate within a solid tumour also possess the ability to adapt and survive in various stress conditions.^10^

In the tumour microenvironment, limited availability of oxygen and nutrients, such as glucose and amino acids, and an accumulation of lactic acid result in the hypoxic and acidic conditions that allow tumour cells to metastasize.^11^ Hypoxia promotes the accumulation of autophagosomes by inducing the expression of autophagy‐related genes, such as Beclin‐1, ATG5, ATG7 and ATG12.^12^ Cancer cells survive the stressful conditions of the tumour microenvironment by adapting to hypoxia through intracellular degradation and autophagy.^13^ In response to hypoxia, tumour cells induce apoptotic‐related proteins such as BNIP3 or TP53 to elicit cell death^14^, ^15^ or express cell survival proteins such as EGFR and BCL2, which are associated with autophagy, to become resistant to chemotherapy.^16^, ^17^, ^18^

Overall gene expression is suppressed under hypoxic conditions because of changes in histone acetylation and methylation.^19^ Therefore, the cellular response to hypoxia is mainly through post‐transcriptional mechanisms involving RNA‐binding proteins (RBPs) and microRNAs (miRNAs).^20^ Changes in transcriptional levels, in turn, lead to the activation of hypoxia‐inducible factor (HIF), which is a heterodimer composed of a hypoxia‐inducible α subunit and a constitutively expressed β subunit 21 that activates genes containing hypoxia‐response elements (HREs).^22^ Certain RBPs bind to hypoxia‐related transcripts to influence the expression of hypoxic genes.^20^ Human antigen R (HuR) and polypyrimidine tract‐binding protein (PTB) are among the RBPs that are thought to interact with hypoxia‐related transcripts during oxidative stress and hypoxia.^23^, ^24^ In response to CoCl_2_, a hypoxia mimetic, PTB binds to the 3′UTR of HIF‐1α, whereas HuR associates with the 5'UTR and promotes the abundance of HIF‐1α and its binding to PTB..^25^ PTBs are known to function by binding to CU‐rich elements.^26^ The PTB binding of CU‐rich sequences in exonic and intronic regions can influence splice site selection by obstructing the definition of exons and introns or preventing the transition from exons to introns.^27^

PTB protein 3 (PTBP3), also known as ROD1, is involved in nonsense‐mediated mRNA decay and functions as a splicing repressor.^28^, ^29^ Recently, PTBP3 has also been found to regulate epithelial‐mesenchymal transition (EMT) in breast cancer.^30^ PTBP3 binds to the 3′UTR mRNA of ZEB1, an EMT regulatory transcription factor, to prevent its degradation. Therefore, overexpression of PTBP3 leads to an induction of EMT through increased levels of ZEB1. In a previous genome‐wide analysis of PTB‐RNA interactions, it was found that ATG12 mRNA 3′UTR contains a PTBP3 binding motif.^28^ ATG12 is known to co‐ordinate basal autophagy, endolysosomal trafficking and exosome release.^31^ In autophagy, ATG12 forms a conjugate with ATG5 to exhibit E3 ligase‐like activity which facilitates the lipidation of the LC3 family.^32^

In this study, we investigate the role of PTBP3 in the development of PDAC and resistance to therapeutics in relation to autophagy. The sensitivity of pancreatic cells to gemcitabine was determined in relation to PTBP3 expression and autophagy inhibition. We also investigated whether PTBP3 expression promoted PDAC proliferation and examined the effects of hypoxia. Finally, we investigated the direct influence of the PTBP3 binding motif on the expression of ATG12 by RNA pull‐down assays.

## 2 MATERIALS AND METHODS

### 2.1 Clinical specimens

The paired human PDAC and adjacent normal pancreas tissues that were used in this study were obtained from patients who underwent surgical resection at Tongren Hospital, Shanghai, China. The study was approved by the Ethical Committee of Tongren Hospital, Shanghai Jiao Tong University School of Medicine, Shanghai, China, and complies with the principles of the Declaration of Helsinki. All patients provided written informed consent.

### 2.2 Survival analysis using GEPIA

We used gene expression profiling interactive analysis (GEPIA, http://gepia.cancerpku.cn/index.html) to analyse whether there was an association between PTBP3 mRNA expression level and the overall survival time of patients. This tool uses Pearson correlation to determine the relationship between a specific set of data in the Cancer Genome Atlas (TCGA) and Genotype‐Tissue Expression (GTEx).

### 2.3 Cell culture

The human pancreatic cancer cell lines used in this study were PANC‐1, BXPC‐3, SW1990 and Capan‐2, and all were obtained from the Shanghai Cell Bank (Type Culture Collection Committee, Chinese Academy of Sciences, China). The human pancreatic ductal epithelial (HPNE) cell line was obtained from the American Type Culture Collection. All cell lines were maintained in a humidified atmosphere (37°C, 5% CO_2_) in RPMI1640 with 10% FBS (Gibco). To replicate hypoxic conditions, cells were cultured in an incubator with an atmosphere of 93% N_2_, 5% CO_2_ and 2% O_2_.

### 2.4 Cell transfection and stable cell line generation

To knockdown PTBP3, shRNA for human PTBP3 was designed and introduced into a GV112 lentiviral expression plasmid (Shanghai Genechem). Two shRNA plasmids (sh1 and sh2) were constructed against different PTBP3 targets, and a scrambled sequence (Scr) was used as a negative control. To overexpress PTBP3, the cDNA of a fragment encoding the full‐length human PTBP3 ORF sequence was amplified by PCR and then cloned into a GV341 lentiviral expression vector (Shanghai Genechem). Lentiviral containing an empty vector was used as a negative control. To create recombinant lentiviruses, Lipofectamine 2000 transfection reagent (Life Technologies) was used to cotransfect HEK293T cells with recombinant expression lentivectors combined with packaged plasmids. After 48‐hours transfection, the viral supernatant was harvested and the viral titre was quantified. Cells were infected with recombinant lentivirus for 48 hours in medium containing 6 μg/mL polybrene (Sigma‐Aldrich). Transfected cell lines were selected with fresh culture medium containing 2 μg/mL puromycin. For the ATG12 rescue assay, ectopic expression of ATG12 plasmid pDsRed1 Atg12 (contain the ATG12 ORF in combination with the 3′‐UTR) was synthesized by GenePharma. Cells were transfected using Lipofectamine 2000 according to the manufacturer's protocol.

### 2.5 Total RNA extraction and quantitative real‐time PCR (qRT‐PCR)

Total RNAs were extracted from pancreatic cancer tissues or cells by TRIzol (Life Technologies), and a SuperScript III cDNA synthesis kit (Life Technologies) was used for reverse transcription. The qRT‐PCR was performed using 2 μL of cDNA samples with SYBR Green PCR Master Mix (Takara) for 40 cycles on an Applied Biosystems ABI Prism 7500 detection system (Life Technologies). Gene expression was quantified by the 2^−ΔΔCt^ method, and β‐actin expression was used to normalize the data.

### 2.6 Immunohistochemistry

Detection by immunohistochemistry (IHC) was performed using 5 μm deparaffinized tissue sections. After blocking endogenous peroxidase activity in 3% (vol/vol) hydrogen peroxide for 10 minutes, slides were immersed for another 10 minutes in 10 mmol/L sodium citrate buffer for antigen retrieval at sub‐boiling temperature. The sections were then incubated with the following primary antibodies: anti‐PTBP3 antibody (Abcam), anti‐ATG12 antibody (Abcam) and anti‐LC3 antibody (Cell Signaling Technology) according to the suppliers’ instructions. The sections were then incubated with a GTVision III Detection System (Gene Tech Co., Ltd). Staining intensity was scored as follows: 0, none; 1, weak; 2, moderate; and 3, strong. The proportion of stained cells was scored as follows: 0, 0%; 1, 1%‐24%; 2, 25%‐49%; 3, 50%‐74%; and 4, 75%‐100%. The final IHC score was the product of the intensity divided by the proportion. Sections were also stained with haematoxylin and eosin (H&E).

### 2.7 Western blotting

Total proteins were extracted from tissue and pancreatic cells using RIPA lysis buffer with 1 mmol/L PMSF (Beyotime Institute of Biotechnology). Protein concentration was determined using a bicinchoninic acid protein assay. Protein samples of an equal quantity (30 μg) were separated by 10% sodium dodecyl sulphate‐polyacrylamide gel electrophoresis (SDS‐PAGE) and then transferred onto PVDF membranes (Amersham) for analysis by immunoblotting. The membranes were first incubated overnight with antibodies specific for PTBP3 (Santa Cruz Biotechnology), LC3B (Santa Cruz Biotechnology), p62 (Abcam), β‐actin or ATG12 (Cell Signaling Technology), and then probed with secondary antibody (Santa Cruz Biotechnology) and visualized with enhanced chemiluminescence (Pierce).

### 2.8 Cell viability assay

To determine chemotherapy resistance and the influence of autophagy, cells (8 × 10^3^) were seeded into 96‐well plates in 100 μL of growth medium and incubated overnight. Cells were then treated with the indicated concentrations of gemcitabine. To inhibit autophagy, cells were preincubated with 3‐MA for 1 hour before treatment. Viability was assessed by incubating cells with 10 μL of MTT (5 mg/mL, Sigma‐Aldrich) for 4 hour at 37°C. Absorbance was then measured at 570 nm with a microplate reader (BioTek), and GraphPad Prism5 was used to calculate IC_50_. All experiments were replicated in triplicate with individual cultures.

### 2.9 Colony formation assay

To determine the ability to form colonies, cells were seeded into 6‐well plates (500 cells/well) and cultured in RPMI‐1640 containing 10% foetal bovine serum. After 14 days, the plates were fixed in 4% paraformaldehyde and then stained by 1% crystal violet. Colonies consisting of more than 50 cells were counted manually under a dissection microscope.

### 2.10 Analysis of GFP‐LC3 immunofluorescence

Pancreatic cancer cells were transfected with a GFP‐LC3 plasmid using Lipofectamine 2000 (Invitrogen). Cells were incubated for 24 hours and then cultured under normoxia or hypoxia for a further 24 hours. GFP‐LC3 immunofluorescent punctate‐structure was observed using a confocal laser scanning microscope. The experiment was replicated at least four times using approximately 100 cells.

### 2.11 RNA pull‐down assays

Human ATG12 mRNA sequences were first amplified by PCR with a T7 promoter sequence added to the 5′ end of the PCR product. The PCR‐amplified products were then used to synthesize biotinylated RNAs using T7 RNA polymerase and biotin‐cytidine triphosphate to create biotin‐cytidine triphosphate‐labelled RNA (MAXIscript Kit; Ambion) following the manufacturer's instructions. Pull‐down assays were performed by incubating purified biotin‐cytidine triphosphate‐labelled RNA with PANC‐1 cell lysates for 1 hour at room temperature. Protein‐biotinylated RNA complexes were isolated using streptavidin‐Sepharose (Sigma‐Aldrich) following the manufacturer's instructions and then analysed by 10% SDS‐PAGE and immunoblotting with anti‐PTBP3 antibody.

### 2.12 Luciferase reporter assay

To construct the full‐length ATG12 3′UTR luciferase reporter plasmids, PCR products were cloned into a psiCheck2 vector (Promega). Cells were transfected with either luciferase reporter plasmids containing ATG12 3′UTR or empty luciferase reporter plasmids using Renilla luciferase as an internal control. After incubation for 24 hours under hypoxic conditions, cells were lysed and reporter activity was assessed using a Dual‐Luciferase Reporter Assay Kit (Promega) following the manufacturer's instructions. Luciferase activities were normalized to Renilla.

### 2.13 Mouse xenograft models

Cells (5 × 10^6^) that were stably transfected by lentiviral particles with PTBP3 expression vectors or PTBP3 shRNA were injected subcutaneously into female BALB/c nude mice (5 to 6 weeks old, Shanghai SLAC Laboratory Animal Co. Ltd.). All mice were housed in air‐filtered pathogen‐free conditions. Tumour growth was monitored with digital calipers every 7 days and plotted against time. The volume of tumours was estimated using the following equation: volume (mm^3^) = (width)^2^ × length/2. Five weeks after implantation, the mice were killed and the tumours were collected, weighed and fixed in 4% paraformaldehyde. Tumour tissues were stained with H&E, and levels of the Ki‐67 proliferation marker were analysed by immunohistochemistry. This experiment was conducted in accordance with the Animal Welfare Guidelines of the Ethical Committee of Shanghai Jiao Tong University School of Medicine.

### 2.14 Statistical analyses

Pearson correlation was used to determine the relationships between PTBP3 and LC3 or PTBP3 and ATG12 expression. Student's *t* test was used to compare two samples, and ANOVA was used for multiple comparisons. Statistical analysis was performed using SPSS17.0 software, and a value of *P* < .05 was considered to have statistical significance. All results are expressed as the mean ± standard deviation (SD) of three or more separate experiments.

## 3 RESULTS

### 3.1 PTBP3 is overexpressed in PDAC

**Figure 1:**
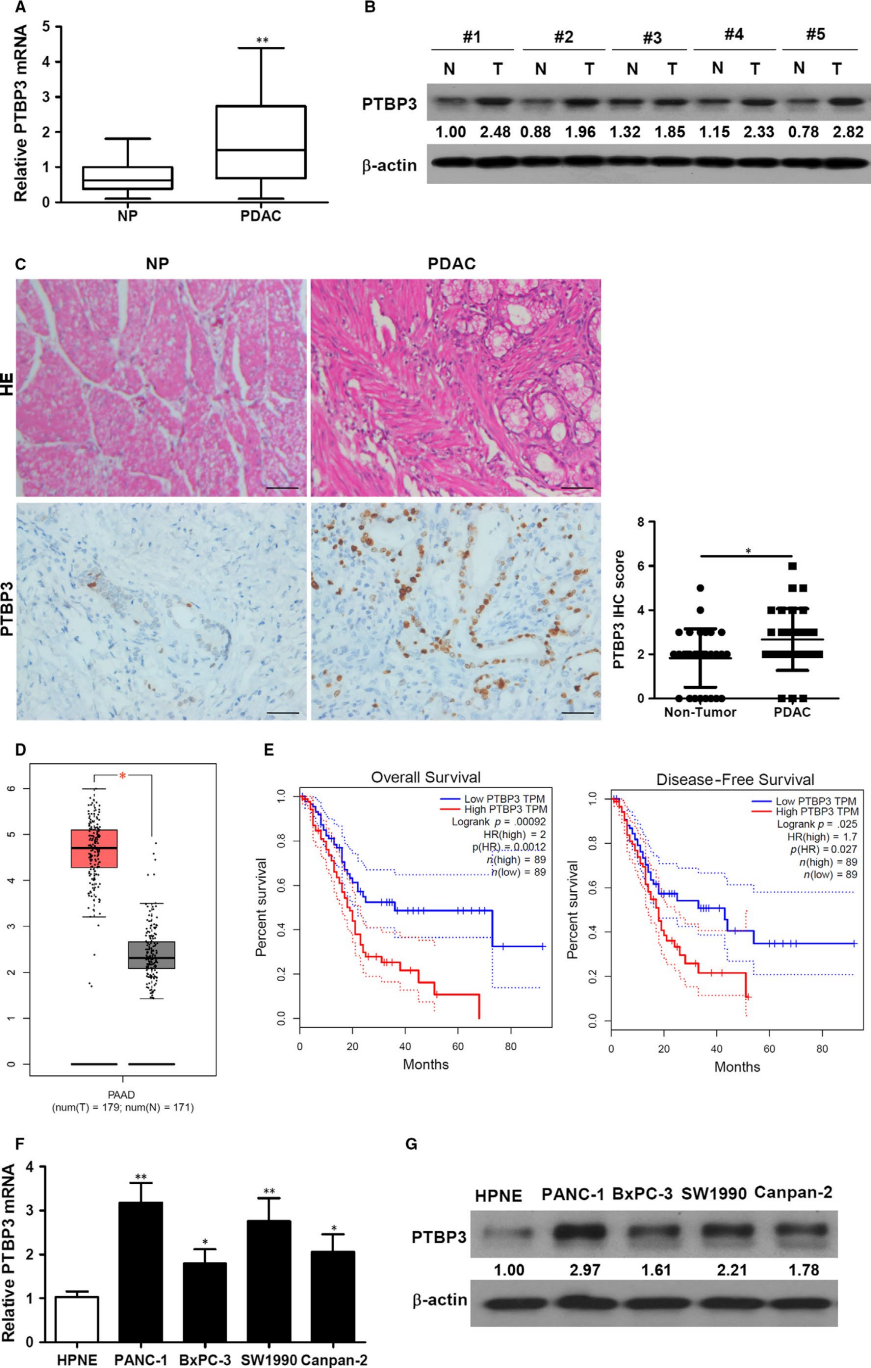
Up‐regulation of PTBP3 in pancreatic ductal adenocarcinoma (PDAC) tissues and cells. A, Relative expression of PTBP3 mRNA in PDAC and matched adjacent non‐tumour tissue (NP) were detected by RT‐qPCR. N = 20. B, Western blot analysis of PTBP3 expression in PDAC (T) and matched adjacent non‐tumour tissue (N) specimens obtained from five PDAC patients. Densitometric quantification of expression is indicated below the lanes and of the corresponding protein relative to β‐actin control. C, Representative images of H&E staining and PTBP3 expression in PDAC and matched adjacent non‐tumour tissue (NP) by immunohistochemical analysis and the IHC score of PTBP3 staining are shown. Scale bar = 50 μm. N = 30. D, PTBP3 mRNA expression in pancreatic adenocarcinoma tissues (T) and normal tissues (N) obtained fromusing GEPIA. E, Kaplan‐Meier survival curve of overall survival and disease‐free survival obtained fromusing GEPIA. F, Expression of PTBP3 mRNA levels in four PDAC cell lines compared to immortal human pancreatic normal epithelial (HPNE) cell line was detected by RT‐qPCR. G, Protein levels of PTBP3 were detected by Western blot analysis. One of three experiments is shown. *P < .05, **P < .01

To determine whether PTBP3 was up‐regulated in PDAC, we analysed its expression in PDAC tissues using RT‐qPCR. The expression of PTBP3 mRNA was found to be significantly higher in PDAC tumour tissue than in matched adjacent non‐tumour tissue (Figure 1A). Western blotting and immunohistochemical (IHC) analysis confirmed that protein levels of PTBP3 were higher in PDAC tumour tissue than in non‐tumour tissue (Figure 1B,C). Further, we analysed the expression of PTBP3 mRNA by using GEPIA confirmed that PTBP3 mRNA expression was higher in PDAC tumour tissue than in non‐tumour tissue (Figure 1D). The relationship between PTBP3 mRNA expression and overall or disease‐free survival revealed that patients with higher expression levels of PTBP3 mRNA exhibited a significantly shorter overall survival time and disease‐free survival time by GEPIA (Figure 1E). Having established that the expression of PTBP3 was higher in PDAC tumour tissue, we next compared mRNA and protein levels of PTBP3 in four different PDAC cell lines (PANC‐1, BxPC‐3, SW1990 and Capan‐2). We used a human pancreatic normal epithelial cell line (HPNE) as a control. PTBP3 expression was higher in all the PDAC cell lines compared to normal pancreatic cells (Figure 1F,G), with the highest expression found in PANC‐1 and SW1990 (*P* < .01).

### 3.2 PTBP3 promotes tumour cell growth in vitro and in vivo

**Figure 2:**
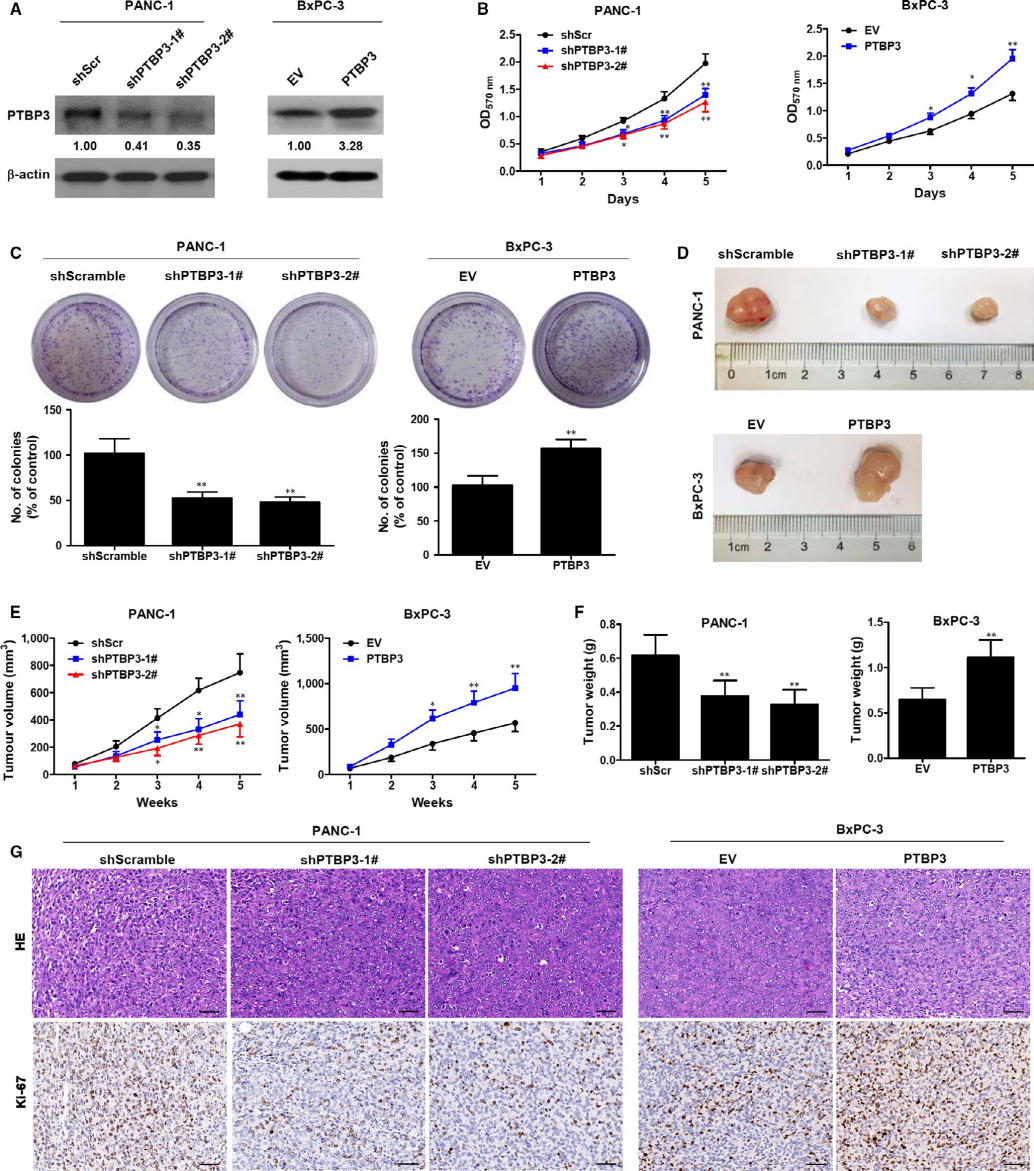
PTBP3 promotes malignancy in pancreatic cancer cells. A, The expression of the PTBP3 protein by Western blot analysis in PANC‐1 cells with a stable knockdown of PTBP3 and BxPC‐3 cells with stable PTBP3 overexpression. B, Cell viability was measured by an MTT assay in PANC‐1 cells with PTBP3 stable knockdown and BxPC‐3 cells with stable PTBP3 overexpression at the indicated times. The numbers below the lanes indicate densitometric quantification of the expression of the corresponding protein relative to β‐actin control. One of three experiments is shown. C, Colony formation assays were performed in PANC‐1 cells with PTBP3 stably knockdown and BxPC‐3 cells with stable PTBP3 overexpression. Statistical analysis showed the percentage of cells that formed colonies relative to the controls (control cells = 100%). *P < .05, **P < .01. D‐F. PANC‐1 cells with a PTBP3 stable knockdown and BxPC‐3 cells with stable PTBP3 overexpression were injected subcutaneously into nude mice (1 × 10^6^ cells per mouse, five mice per group). Representative images of tumours (D), tumour growth curves (E) and tumour weights (F) are shown. G, Representative images of H&E staining and Ki‐67 expression in tumours. Scale bar = 50 μm

To determine whether PTBP3 influences the malignancy of pancreatic cancer cells, we assessed whether the level of PTBP3 expression could alter proliferation by blocking the expression of PTBP3 in PANC‐1 cells and overexpressing PTBP3 in BxPC‐3 cells (Figure 2A). Cell viability, measured by using an MTT assay, was found to be significantly reduced in PANC‐1 cells with a PTBP3 knockdown (*P* < .01) and significantly increased in BxPC‐3 cells overexpressing PTBP3 (*P* < .01) (Figure 2B). Similar results were found in colony formation assays (Figure 2C). The underexpression of PTBP3 significantly reduced the number of colonies, whereas overexpression increased the formation of colonies. To assess whether results in vitro could be replicated in vivo, PANC‐1 and BxPC‐3 cells with PTBP3 underexpressed and overexpressed, respectively, were injected subcutaneously into nude mice. Representative images of tumours are shown in Figure 2D. Tumour growth curves and weights were significantly increased when PTBP3 was overexpressed; however, the opposite occurred when the expression of PTBP3 was blocked (Figure 2E,F). The expression of Ki‐67 was significantly reduced in xenograft tumours treated with PTBP3 shRNA, whereas PTBP3 overexpression promoted Ki‐67 expression in xenograft tumours (Figure 2G). These results indicate that PTBP3 promotes the malignancy of pancreatic cancer cells.

### 3.3 PTBP3 is induced under hypoxia and involved in hypoxia‐induced resistance to gemcitabine in human pancreatic cancer cells

**Figure 3:**
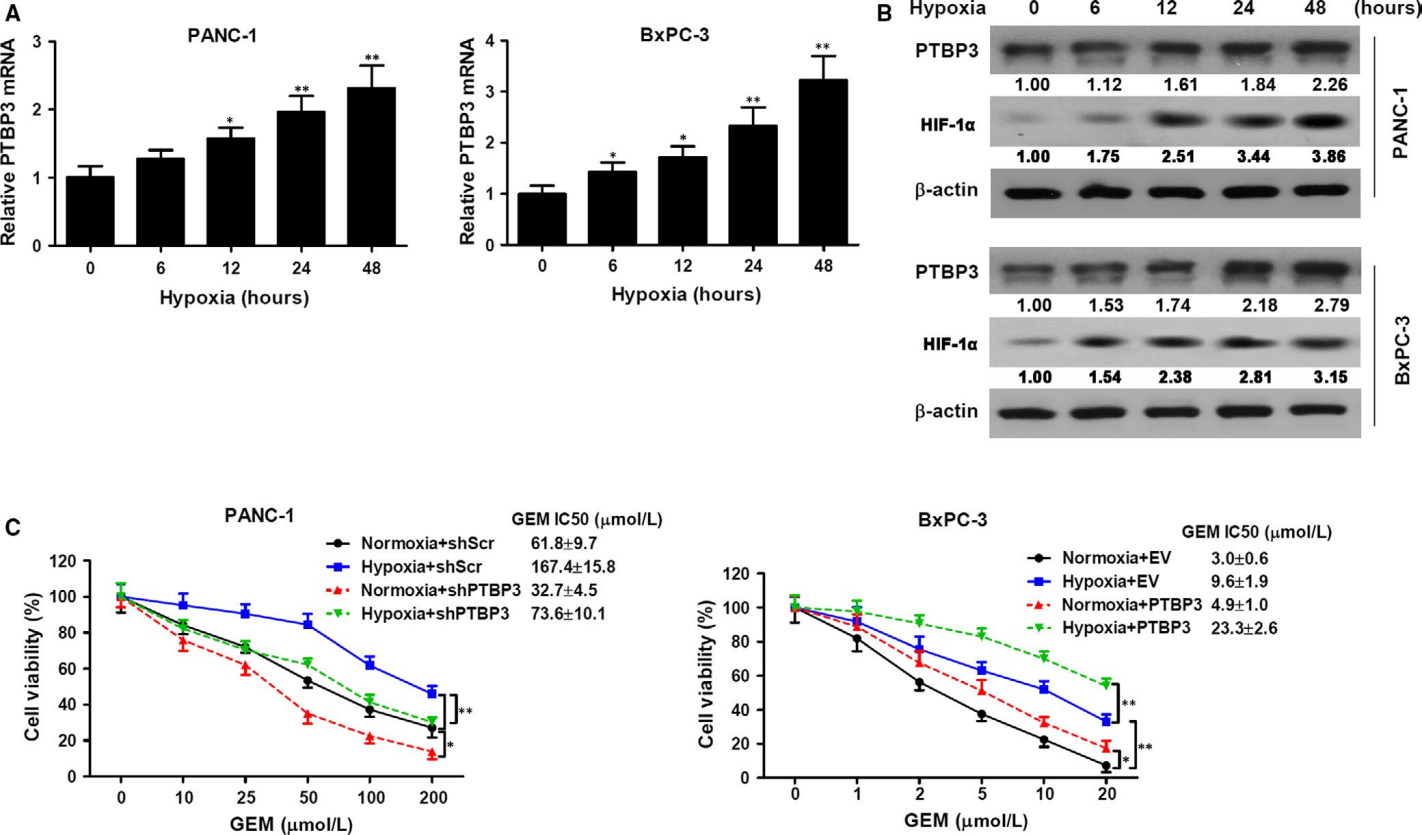
Hypoxic induction of PTBP3 in human pancreatic cancer cells. A, PANC‐1 and BxPC‐3 cells were exposed to hypoxia for the indicated time periods, and mRNA levels were analysed by RT‐qPCR. B, PANC‐1 and BxPC‐3 cells were incubated under hypoxia for the indicated time periods, and protein levels were analysed by Western blotting. C, PANC‐1 cells were transfected with shScr or shPTBP3 (shPTBP3‐2#), and BxPC‐3 cells were transfected with empty vector (EV) or PTBP3 then treated with gemcitabine (GEM) at the indicated concentrations for 48 h under normoxic or hypoxic conditions. Cell viability (mean ± SD, n = 4) was measured using an MTT assay, and IC_50_ values were calculated using GraphPad. *P < .05, **P < .01

To determine whether hypoxia influenced PTBP3 expression in human pancreatic cancer cells, mRNA levels of PTBP3 in PANC‐1 and BxPC‐3 cells exposed to hypoxia were analysed by RT‐qPCR and Western blotting (Figure 3A,B). Hypoxia significantly increased the expression of HIF‐1α, as an intrinsic marker of hypoxia, and PTBP3 in PANC‐1 and BxPC‐3 cells (both *P* < .01). The effects of hypoxia on gemcitabine resistance in pancreatic cancer cells underexpressing or overexpressing PTBP3 were then analysed. As shown in Figure 3C, resistance to gemcitabine was increased under hypoxia in the PANC‐1 and BxPC‐3 cells (*P* < .01). PTBP3 silencing resulted in increased sensitivity to gemcitabine of PANC‐1 cells under normoxic conditions (*P* < .05) and abolished hypoxia‐induced gemcitabine resistance (*P* < .01), whereas PTBP3 overexpression promoted gemcitabine resistance in BxPC‐3 cells under normoxic conditions (*P* < .05) and significantly enhanced hypoxia‐induced gemcitabine resistance (*P* < .01). Overall, these results indicate that PTBP3 is induced in human pancreatic cancer cells under hypoxia and is involved in hypoxia‐induced resistance to gemcitabine.

### 3.4 PTBP3 promotes autophagy in response to hypoxic stress

**Figure 4:**
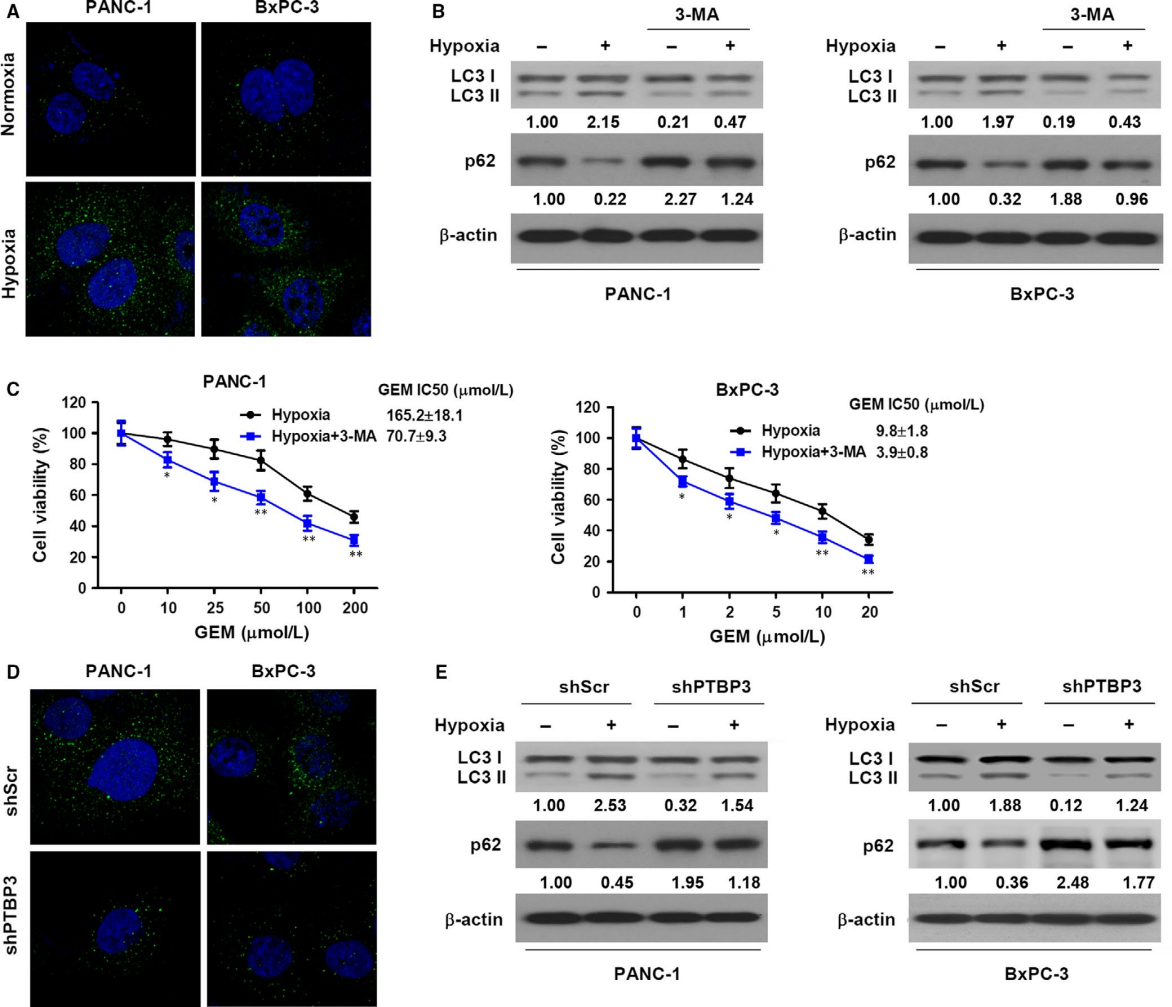
PTBP3 enhances autophagy in hypoxia. A, Representative images of autophagosome formation upon hypoxia. GFP‐LC3 was transfected into PC cells for 24 h, and then, cells were exposed to hypoxia for 24 h. Images were taken by a confocal laser scanning microscope. B, Pancreatic cancer cells were exposed to normoxia or hypoxia (2%) with or without 3‐MA (50 μmol/L) for 24 h. Levels of LC3B and p62 were determined by Western blotting. The numbers below the lanes indicate densitometric quantification of the expression of the corresponding protein relative to β‐actin control. The numbers below the LC3 lane indicate the ratio of LC3‐II to LC3‐I. C, One of three experiments is shown. Pancreatic cancer cells were treated with gemcitabine (GEM) at the indicated concentrations for 48 h under normoxic or hypoxic conditions with or without 3‐MA (50 μmol/L). Cell viability (mean ± SD, n = 4) was measured using an MTT assay, and IC_50_ values were calculated using GraphPad. *P < .05, **P < .01. D, Pancreatic cancer cells with a stable knockdown of PTBP3 were transfected with GFP‐tagged LC3. After 24‐h transfection, cells were incubated under hypoxia for 24 h. Representative images showed the punctate GFP‐LC3 taken by confocal laser scanning microscope. E, Stable knockdown of PTBP3 in PC cells incubated under normoxia or hypoxia for 24 h. LC3B and p62 were determined by Western blotting. The numbers below the lanes indicate densitometric quantification of the expression of the corresponding protein relative to β‐actin control. The numbers below the LC3 lane indicate the ratio of LC3‐II to LC3‐I. One of three experiments is shown

To investigate whether autophagy may be involved in PTBP3 enhanced hypoxia‐induced gemcitabine resistance, we assessed autophagosome formation upon hypoxia and the corresponding levels of autophagy‐related markers. The formation of autophagosomes is indicated by the conversion of LC3 from a soluble form, LC3‐I, to a lipid‐bound form, LC3‐II, and a reduction in the levels of p62.^33^ During autophagy, LC3‐I becomes conjugated to phosphatidyl‐ethanolamine and recruited as LC3‐II to autophagosomal membranes, whereas p62, a ubiquitin‐binding protein that interacts with LC3, is degraded. Images taken by confocal laser scanning microscopy of pancreatic cancer cells (PANC‐1 and BxPC‐3) transfected with GFP‐tagged LC3 showed that the level of membrane‐bound LC3 increased in cells under hypoxic conditions compared with normoxic conditions (Figure 4A), indicating an increase in the level of autophagy. PANC‐1 and BxPC‐3 cells were also exposed to normoxia or hypoxia (1%) in the presence of 3‐methyladenine (MA), an autophagic inhibitor. The ratio of LC3‐II to LC3‐I as measured by Western blotting was higher in cells grown under hypoxic conditions but lower in cells grown with 3‐MA (Figure 4B), confirming an increase in autophagy. Viability was assessed in PANC‐1 and BxPC‐3 cells treated with gemcitabine under hypoxic conditions with or without 3‐MA. For both cell lines, cell viability was reduced when autophagy was inhibited (Figure 4C), which indicates that autophagy may play a role in hypoxia‐induced gemcitabine resistance. When PTBP3 is knocked down in both cell lines under hypoxic conditions, confocal laser scanning microscopy showed that the levels of GFP‐tagged LC3 decreased (Figure 4D). Moreover, LC3‐II and p62 levels were determined by Western blotting and indicated that autophagy was lower in PANC‐1 and BxPC‐3 cells when PTBP3 was silenced (Figure 4E). These results indicate that PTBP3 enhances autophagy in pancreatic cancer cells grown under hypoxia.

### 3.5 PTBP3 binds to ATG12 3′‐UTR and regulates ATG12 expression

**Figure 5:**
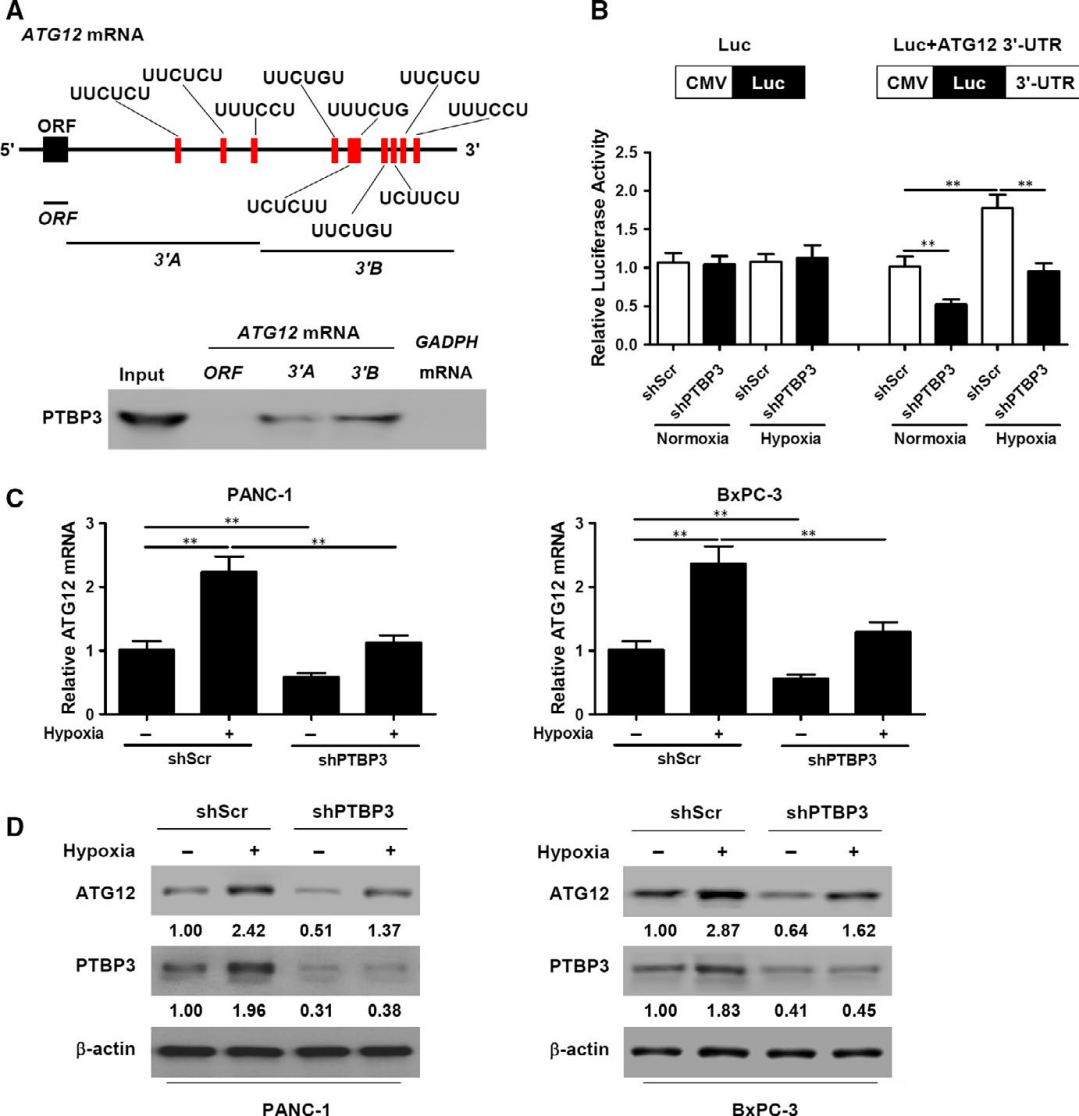
PTBP3 regulates ATG12 expression in response to hypoxic stress. A, Upper panel, schematic of human ATG12 mRNA with PTBP3 binding motifs. Lower panel, RNA pull‐downs. The biotinylated open reading frame (ORF) or 3′UTR (3′A and 3′B) mRNA transcripts synthesized and incubated with PANC‐1 cell lysates, and the interaction between PTBP3 and biotinylated transcripts was analysed by Western blotting. B, PANC‐1 cells with a stable knockdown of PTBP3 were transfected with luciferase reporter constructs (luciferase control or fused with full‐length ATG12 3′UTR) and subjected to hypoxia or normoxia for 24 h. Renilla luciferase activity was normalized to firefly luciferase activity and is the average of three experiments ± SEM. (C and D) Pancreatic cancer cells with a stable knockdown of PTBP3 were incubated in normoxia or hypoxia for 24 h. ATG12 mRNA was assayed by RT‐qPCR (C) ATG12 and PTBP3 protein expression was assayed by Western blot (D) The numbers below the lanes indicate densitometric quantification of the expression of the corresponding protein relative to β‐actin control. **P < .01

PTBs are known to function by binding to CU‐rich elements,^28^ computational predictions indicated that human ATG12 3′‐UTR contains PTBP3 binding motifs (Figure 5A). We divided ATG12 3′‐UTR into two transcripts, A and B, and confirmed that they bound with PTBP3 through RNA pull‐downs. The biotinylated open reading frame (ORF) of ATG12 or the two 3′‐UTR (3′A and 3′B) mRNA transcripts was synthesized and incubated with PANC‐1 cell lysates. Western blot analysis confirmed that PTBP3 could bind to both the 3′A and 3′B mRNA transcripts but not the ORF of ATG12 (Figure 5A). A luciferase reporter assay was used to gain more information about the interaction between PTBP3 and the 3′‐UTR of ATG12. PANC‐1 cells containing a stable knockdown of PTBP3 were transfected with luciferase reporter constructs (luciferase control or fused with full‐length ATG12 3′‐UTR) and subjected to normoxia or hypoxia. As shown in Figure 5B, PTBP3 knockdown reduced luciferase activity in PANC‐1 cells transfected with full‐length ATG12 3′‐UTR under normoxia. Luciferase activity was strongly induced in hypoxic conditions in PANC‐1 cells, which was completely abolished when PTBP3 was silenced. The relative levels of ATG12 mRNA were then determined in PANC‐1 and BxPC‐3 cells with a stable knockdown of PTBP3 after incubation in normoxia or hypoxia (Figure 5C,D). Hypoxia increased levels of ATG12 in PANC‐1 and BxPC‐3 cells. PTBP3 knockdown resulted in attenuated ATG12 mRNA and protein expression in normoxia and completely abrogated hypoxia‐mediated induction of ATG12, both at the mRNA and protein level. Overall, the results indicate that PTBP3 post‐transcriptionally regulates ATG12 expression in response to hypoxic stress.

### 3.6 Regulation of autophagy and chemoresistance by PTBP3 is ATG12 dependent

**Figure 6:**
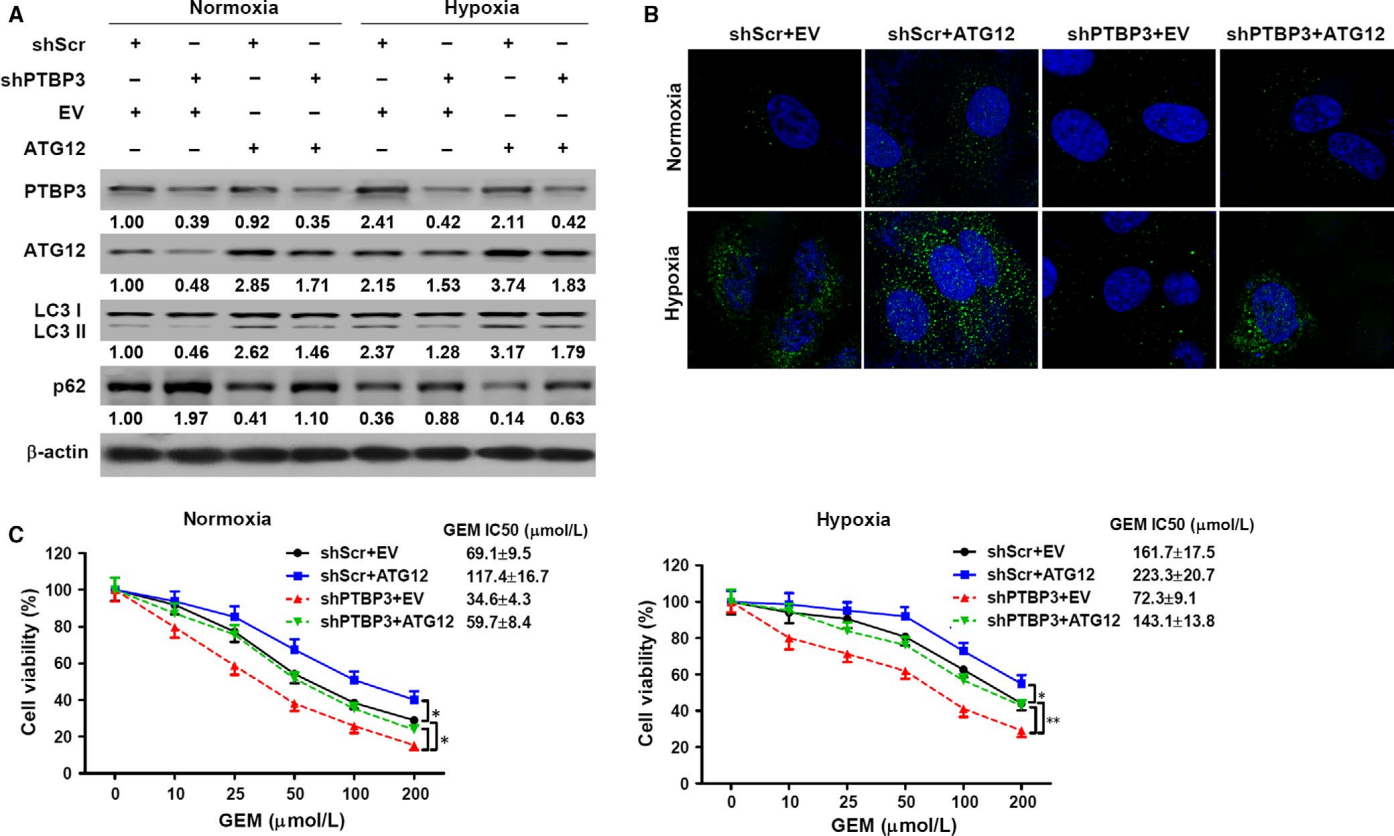
Regulation of autophagy and chemoresistance by PTBP3 is mediated by ATG12. A, PANC‐1 cells with a stable knockdown of PTBP3 were transfected with EV or ATG12 and subjected to hypoxia or normoxia for 24 h. ATG12, PTBP3, LC3B and p62 were determined by Western blot analysis. The numbers below the lanes indicate densitometric quantification of the expression of the corresponding protein relative to β‐actin control. The numbers below the LC3 lane indicate the ratio of LC3‐II to LC3‐I. B, PANC‐1 cells with a stable knockdown of PTBP3 were cotransfected with empty vector (EV) or ATG12 and GFP‐LC3 and subjected to hypoxia or normoxia for 24 h. Representative images showed the punctate GFP‐LC3 were taken by confocal laser scanning microscope. C, PANC‐1 cells with a stable knockdown of PTBP3 were transfected with EV or ATG12 for 24 h and then treated with gemcitabine (GEM) at the indicated concentrations for 48 h under normoxic or hypoxic conditions. Cell viability (mean ± SD, n = 4) was measured using an MTT assay, and IC_50_ values were calculated using GraphPad. *P < .05, **P < .01

To further investigate whether the expression of ATG12 could influence autophagy and subsequently chemoresistance, PANC‐1 cells with a knockdown of PTBP3 were transfected with empty vector (EV) or ATG12 and subjected to hypoxia or normoxia; levels of ATG12, PTBP3, LC3‐II and p62 were then determined. The highest levels of ATG12 and PTBP3, highest LC3‐II ratio and the lowest levels of p62 were found in cells overexpressing ATG12 under hypoxic conditions (Figure 6A). Knockdown of PTBP3 decreased LC3‐II ratio and increased p62 levels under normoxia, overexpressing ATG12 attenuated PTBP3 knockdown‐mediated reduction of LC3‐II ratio and induction of p62, both under normoxia and hypoxic conditions. Reduction of autophagy by PTBP3 knockdown was further confirmed by a decrease in the number of autophagosomes visualized via transient LC3‐GFP transfection, especially in cells silencing PTBP3 under hypoxia, and this is attenuated by ATG12 overexpression (Figure 6B). PANC‐1 cells overexpressing ATG12 were then treated with gemcitabine under normoxic or hypoxic conditions. Cell viability was significantly higher when ATG12 is overexpressed in normoxic and hypoxic conditions (IC_50_ 117.4 ± 16.7, 223.3 ± 20.7 μmol/L, respectively) (Figure 6C). Knockdown of PTBP3 increased gemcitabine sensitivity, whereas ATG12 overexpression decreased gemcitabine sensitivity and attenuated PTBP3 knockdown‐mediated sensitivity to gemcitabine. Overall, these results indicate that the regulation of autophagy and chemoresistance by PTBP3 is mediated by ATG12.

**Figure 7:**
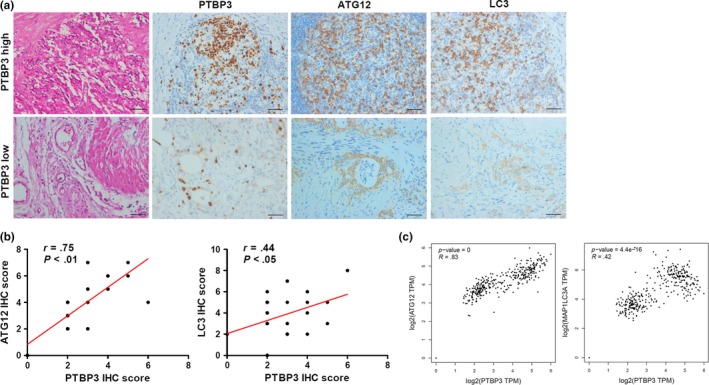
The relationship between PTBP3 and ATG12 in the clinical samples. A, Representative images of H&E staining and PTBP3, ATG12 and LC3B expression in tumour sections of 30 patients with PDAC. Scale bar = 50 μm. B, Pearson correction of PTBP3 and ATG12 (n = 30), PTBP3 and LC3 (n = 30). C, Correlation in PTBP3, ATG12, LC3 (MAP1LC3A) expression obtained using GEPIA

Subsequently, IHC analysis of ATG12 and LC3 expression was performed using the same PDAC specimens that were used for PTBP3 staining. On the basis of immunostaining for PTBP3, ATG12 and LC3, we observed a close correlation between the protein expression levels for PTBP3 and ATG12, PTBP3 and LC3, respectively. The tumour sections of patients with high levels of PTBP3 also have high levels of ATG12 and LC3‐II (Figure 7A). The Pearson correlation between PTBP3 and ATG12 (*P* < .01) and PTBP3 and LC3 (*P* < .05) IHC scores is significant (Figure 7B). In addition, we analysed the correlation between PTBP3, ATG12 and LC3 expression by using GEPIA, and the results indicated that PTBP3 had a strong correlation with the expression of ATG12 and LC3 in pancreatic adenocarcinoma (Figure 7C). Confirming that in human PDAC tissue, PTBP3 expression and ATG12 levels are correlated.

## 4 DISCUSSION

An association between hypoxia‐induced chemoresistance and autophagy is becoming increasingly apparent.^34^, ^35^, ^36^ Moreover, hypoxia‐associated genes and autophagy are implicated with a less favourable outcome in PDAC.^37^, ^38^ PTBs are known to regulate gene expression by binding to hypoxia‐related transcripts 20; therefore, we also studied the role of PTBP3 in the development of PDAC and therapeutic resistance in tumour tissue and in pancreatic cancer cell lines under hypoxic stress. In addition, we determined whether PTBP3 influenced the stress response against hypoxia and the cytotoxic effects of gemcitabine and investigated whether PTBP3 expression promoted the proliferation of PDAC cells and its direct influence on the expression of ATG12. ATG12 is a protein that is known to regulate autophagy.^31^ Therefore, we also assessed the sensitivity of pancreatic cancer cells to gemcitabine in relation to ATG12 expression and autophagy inhibition.

We found that PTBP3 is overexpressed in PDAC and that it can bind to the ATG12 3′‐UTR and regulate ATG12 expression. PTBP3 was able to promote tumour cell growth in vitro and in a xenograft mouse model in vivo. Other studies have found that PTBP3 is associated with chemoresistance, and the inhibition of PTBP3 in gastric cancer cells induces apoptosis and cell cycle arrest and increases sensitivity to 5‐fluorouracil.^39^ In gastric cancer, PTBP3 is thought to contribute to metastasis through the alternative splicing of Caveolin 1 (CAV1), an integral membrane protein with two isoforms (CAV1α and CAV1β) that have opposing roles in cell survival.^40^ In our study, PTBP3 is induced under hypoxia and involved in hypoxia‐induced resistance to gemcitabine in human pancreatic cancer cells. Previously, PTBP3 was found to bind with miR‐210, which is involved in endothelial cell adaptation to hypoxia.^41^ PTBP3 was inhibited by miR‐210, and both PTBP3 mRNA and protein were down‐regulated under hypoxia in a miR‐210‐dependent manner. However, when PTBP3 was overexpressed, hypoxia‐induced cell death was significantly increased. The authors proposed that PTBP3 expression regulates cell survival and that its repression is involved in miR‐210 anti‐apoptotic function.^41^

We found that PTBP3‐mediated the regulation of autophagy and chemoresistance by regulating ATG12 mRNA stability. Other studies have found that the regulation of autophagy through the expression of ATG12 increases the sensitivity of cancer to therapeutics,^42^, ^43^ and the inhibition of autophagy can significantly enhance the results of combination therapy.^44^ Sun et al^43^ found that autophagy was associated with increased sensitivity to epirubicin (EPI) and that the expression of autophagy/Beclin 1 regulator 1 (Ambra1) was negatively correlated with EPI sensitivity in breast cancer cells. Moreover, ATG12 was found to be a key regulator of Ambra1 and EPI‐induced autophagy. In an earlier study on chemoresistance in lung adenocarcinoma, miR‐200b was reported to regulate autophagy in docetaxel resistance by interacting with ATG12.^42^ MiR‐200b‐dependent ATG12 down‐regulation could inhibit autophagy and enhance the chemosensitivity of lung adenocarcinoma cells both in vivo and in vitro. Similarly, miR‐29c overexpression was reported to enhance the effect of gemcitabine by reducing autophagy in pancreatic cancer cells.^45^ MiR‐29c is proposed to interact with ubiquitin‐specific peptidase (USP)‐22, a deubiquitinating enzyme known to induce autophagy and promote pancreatic cancer cell survival. In the present study, we also found that down‐regulating ATG12 by reducing the expression of PTBP3 inhibited autophagy and increased sensitivity to gemcitabine in pancreatic cancer cells. In our results, the levels of PTBP3 strongly reflect those of ATG12 and suggest that PTBP3 regulates ATG12 expression in response to hypoxic stress. More importantly, increased ATG12 expression was associated with an elevated level of autophagy and resistance to gemcitabine. Whereas low levels of ATG12 were associated with less autophagy and enhanced sensitivity to gemcitabine, in a recent study, the inhibition of endoplasmic reticulum stress‐mediated autophagy was able to enhance the effectiveness of gemcitabine in PDAC.^38^ In agreement with our results, the anti‐proliferative effect of gemcitabine was significantly increased when autophagy was inhibited. Thakur et al^38^ found that the addition of sunitinib or chloroquine to gemcitabine significantly increased survival in an animal pancreatic cancer model without an increase in toxicity. The authors proposed that sunitinib and chloroquine can reduce tumour growth through the suppression of autophagy and increased apoptosis.

Increasing evidence suggests an association between hypoxia and chemoresistance in cancer cells, in particular, through the involvement of HIF‑1α.^35^, ^36^ Hypoxia was found to inhibit cisplatin‐induced apoptosis in ovarian cancer cells and enhance chemoresistance to cisplatin.^35^ In bladder cancer cells, hypoxia was found to activate autophagy and significantly reduce gemcitabine‐induced apoptosis compared with normoxic conditions.^36^ In agreement with our findings, gemcitabine cytotoxicity was increased in bladder cancer cells when combined with the autophagy inhibitor 3‐MA. Hypoxia stimulates translation of HIF‐1α and HIF‐2α proteins by distributing HIF‐α mRNAs to larger polysome fractions. This translational control elevates the levels of HIF‐1α by 40 to 50% during short‐term hypoxia.^46^ Autophagy has been found to mediate cisplatin resistance under hypoxia in lung cancer cells.^34^ Similar to our findings, when autophagy was inhibited by 3‐MA or a siRNA targeting an autophagy‐related gene (ATG5), sensitivity to cisplatin increased. Wu et al^34^ propose that a more efficient autophagic process under hypoxia increased cancer cell survival. Tumour hypoxia has also been found to increase breast cancer cell survival through NEAT1, which is a long non‐coding RNA (lncRNA) regulated principally by HIF‐2α rather than by HIF‐1α.^47^ Induction of NEAT1 by hypoxia leads to accelerated proliferation, improved survival and reduced apoptosis of breast cancer cells.^48^ Interestingly, PTBP3 has been implicated in the promotion of hepatocellular carcinoma by disrupting the splicing balance of NEAT1.^49^ The PTBP3 protein was found to recruit abundant lnc‐NEAT1 splicing variants and precursors of miR‐612 in the nucleus. Therefore, it could be possible that the up‐regulation of PTBP3 leads to an imbalance of non‐coding transcriptional response in cancer cells. More recently, in addition to its role in hypoxia, NEAT1 has been associated with autophagy.^50^

The present study proposes that PTBP3 is up‐regulated in PDAC and regulates the expression of ATG12 post‐transcriptionally in response to hypoxia by binding to CU‐rich elements in the ATG12 3′‐UTR. ATG12 up‐regulation signifies an increased level of autophagy and resistance to chemotherapeutics. Therefore, targeting the expression of PTBP3 under hypoxia could influence the response to therapy in PDAC.

## CONFLICT OF INTEREST

The authors confirm that there are no conflicts of interest.

## AUTHOR CONTRIBUTIONS

Shang MY and Wang ZM conceived and designed the experiments; Ma J, Weng L, Jia YP and Liu BY performed the experiments; Wu SQ, Xue L, Yin X and Mao AW analysed the data; Ma J, Weng L, Shang MY and Wang ZM wrote the paper. All authors read and approved the final manuscript.

## Data Availability

The data that support the findings of this study are available from the corresponding author upon reasonable request.
